# Integrating case reports into systematic reviews: methodological strategies and challenges

**DOI:** 10.1186/s13643-025-02955-4

**Published:** 2025-10-31

**Authors:** Mohammad J. J. Taha, Mohammad T. Abuawwad

**Affiliations:** Kasr-Alainy Faculty of Medicine, Cairo University, Cairo, Egypt

**Keywords:** Case reports, Case series, Evidence-based medicine, Methodology, Systematic review

## Abstract

Systematic reviews are used in medical research to synthesize evidence by collecting evidence from available literature without overlooking important sources or pieces. Several guidelines are available for the methodological implementation of a systematic review; however, none of them include case reports or case series as a potential source of information that could be pooled and analyzed. Although case reports may be criticized as an evidence source, they are important for documenting certain types of evidence such as rare conditions, adverse events, or novel emergent diseases. In this article, we attempt to set the foundation for a guideline on the systematic review of cases, and we suggest a step-by-step guide for investigators to employ for systematically reviewing case reports. We also dissect the application of this methodology in the available literature and review a case study of a systematic review of case reports. Generally, case reports can be synthesized into a systematic review, providing evidence on matters that may be hard to investigate using other methodologies, but concerns regarding quality and level of evidence remain present.

## Introduction

Systematic reviews (SR) aim at pooling evidence from primary research articles in a systematic manner. The systematic aspect focuses on performing comprehensive searches to reduce the chances of missing articles or evidence that may be of importance. Pooling of evidence allows for parallel comparisons and evaluation, and preferably modeling and regression, yielding conclusions that may be generalized further than actual observation, at least theoretically, in addition to the conclusive evidence regarding the extent or effect of observed measurements. Systematization of the search process went through several phases of development and improvement, until 1976 when Gene V Glass published his famous article on Primary, Secondary, and Meta-Analysis of Research [1]; the introduction of meta-analysis of evidence highlighted the need for a systematic manner of evidence collection, especially in clinical research. The 1990 s featured the establishment of organizations of pivotal importance in systematizing research, like The Cochrane Collaboration in 1993 and the Campbell Collaboration in the year 2000 [2, 3]. Later on, came the introduction of guidelines such as Preferred Reporting Items for Systematic Reviews and Meta-Analyses (PRISMA) which is often used by researchers today [4]. Case reports and case series are two types of observational studies that report on rare diseases or uncommon adverse effects. Drawing evidence-based medicine from a case report is difficult because it only reports on one case, and in rare diseases, alternative observational studies such as cohort or case–control studies cannot be undertaken due to the rarity of the patients. So, collecting case reports and case series on a rare disease or a specific complication of therapy or disease in a systematic manner can help in the development of evidence-based medicine in this particular field. This article aims to introduce the concept of SRs of cases, in a critical view, in order to investigate the strengths and weaknesses of this methodology, and suggest steps towards achieving the best medical evidence-based reviews.

## Problem statement: why are case reports/series not included in SR methods?

Several standardized methodologies for conducting a SR are present, the most famous of which is the PRISMA, Cochrane handbook, Meta-analyses Of Observational Studies in Epidemiology (MOOSE) guideline, A Measurement Tool to Assess systematic Reviews (AMSTAR), JBI’s Manual for Evidence Synthesis, the Grading of Recommendations Development, Assessment and Evaluation (GRADE), and Enhancing the Quality and Transparency Of health Research (EQUATOR) [4–10]. None of these guidelines include the use of case reports or case series as the primary included study design in a SR, except for JBI’s, which mentions case reports as a possible type of study to include in the inclusion criteria; however, the guideline does not expand on how to synthesize evidence from this study type. Systematic reviews usually exclude case reports and case series, which is attributed to the absence of contralateral controls, introducing a high risk of bias [11]. Risk in case reports or case series can be introduced by lack of randomization, blinding, and most importantly, potential selective reporting bias. Another important aspect is the presence of a large gray area of confounding in case reports.

Synthesizing a standardized case reporting guideline is a difficult task, since cases vary significantly in their clinical characteristics, extent of involved systems, among several other aspects; nevertheless, almost all clinical cases share a common outline that can be put into a standardized manner, reducing the room for variation and introducing some controlling measures to the internal validity. Such standardization was attempted by the Case Reporting Guidelines organization, which introduced [12] as a checklist guide to reporting case reports [13]. The CARE guidelines require the collection of information regarding patient information, clinical findings, timeline, diagnostic assessment, therapeutic intervention, and follow-up and outcomes; thus, it outlines the important items that must be reported in a case report, with subheadings describing the components of each item. This could help improve case reporting into a guided conventional data presentation, helping reduce selective bias and confounding due to missing or under-reported history or baseline information.

## Why we need to include case report/series in SRs, and what type of information we expect to attain by this methodology? And at what level of evidence?

To understand the type of evidence that could be generated from a SR of cases, we first need to appreciate the type of evidence these cases provide. Case reports and case series provide evidence that may not be available in other research designs, especially in clinical conditions of great variety, rarity, or novelty, where enough homogenous information that is coherent is hard to come by. Examples include rare or severe fungal and parasitic infections [14–16], adverse events of extreme conditions [17–20], congenital anomalies [21–23], traumatic complications [24, 25], or novel emergent conditions like the COVID-19 pandemic [26–31]. In addition, in emerging therapies or techniques that are not yet heavily investigated, and for which evidence like RCTs or large cohort studies is either lacking or limited in number and does not suffice the minimum requirement for SRing or meta-analysis [6]. Similarly, some conditions or management modalities are difficult to investigate by RCT or cohort methodologies, like specific surgical complications or unexpected surgical or medical outcomes. In similar conditions, where stronger evidence does not allow for a SR, collecting and reviewing available case reports could constitute the grounds for higher-level investigation targeted towards specific aspects of the disease or management. Therefore, the need for evaluating cross-case evidence in these conditions and many others remains present, particularly since alternative study designs with higher evidence levels are often absent for such conditions. In general, evidence from SRs of cases can fall within one of four main types:

1. Descriptive evidence
  A collection of cases with sufficient information can provide clinical description with great detail on patients sociodemographic, presentation details and outcome of rare diseases or conditions.
2. Hypothesis-generating
  A SR of case reports can lay the foundation for higher level evidence by establishing preliminary evidence, and by directing hypothesis generation process, providing an outline for future preference. Also, SR of case reports can study the relations between specific outcome of the patients and their associated factors which may answer the question, why this patient is associated with bad complications or poor prognosis?
3. Preliminary safety and efficacy
  Similar to rare conditions, novel treatments lack sufficient data for higher level evidence, therefore; preliminary evidence on efficacy and more importantly safety can be established using a SR of cases. This type of evidence is highly needed for rare and diverse adverse events. Also, helping in finding preliminary evidence about the effective treatment and management of the disease will guide the future intervention study to investigate the most effective management with the lowest side effects, which reduces the risks and improves the quality of the randomized clinical trials.
4. Clinical insights
  Clinicians often benefit from insight from cases with adjacent clinical presentation, and passing clinical experiences in a systematic evidence-based manner can be achieved with the help of a SR of cases.

A clear level of evidence for SRs of cases is hard to determine; nevertheless, in accordance with almost all evidence-based medicine hierarchies, case reports and case series fall within levels IV to V. Since controlled evidence is often classified as level IV or III evidence, a SR of cases would be only as high as level IV [32]. From this point up in the level of evidence, a SR of cases could pave the way for a higher-level evidence investigation. For example, following the publication of a SR of cases of Guillain–Barre syndrome in association with COVID-19 vaccination [28], researchers began collecting and generating higher-level evidence, arriving at cohort studies, SRs, and meta-analyses of cohort studies [33]. This later generated evidence is at level III of evidence, meaning that a climb in the level of evidence had occurred, starting with SR of cases [32]. In comparison to SR of cohort studies, SR of case reports showed that AstraZeneca (Adenovirus-vectored) was associated with a higher risk of GBS; similarly, SR of cohort studies reported that AstraZeneca vaccines showed a 2.4 times increased risk of GBS compared with mRNA-based vaccines. Here we can conclude that the SR of cohort studies gives similar broad results to the SR of case reports, but with more detailed results.

### Limitations and risk of bias

The major issues with SRs of cases were the causal link behind their exclusion from guidelines for performing SRs. These concerns could easily hinder the internal and external validity, and they must be considered when performing, validating, or reading a SR of cases. A SR of case reports would naturally be subject to errors due to bias issues, generalizability restrictions, and difficulties in quality assessment.

Three major sources of bias are easily incorporated in any case report or case series, namely: selection, randomization, and reporting bias. Due to a lack of controls, it is often difficult to draw a clear line as to where cases fall in contrast with controls; therefore, case series feature cases of certain characteristics, making them biased in patient selection and randomization. Reporting bias can also be easily introduced into a case report or a case series since authors can be tempted to report only extraordinary findings and under-report general or common features. Case reports can be prone to a high risk of bias if authors refrain from providing enough information supporting their diagnosis, management plan, or overall outcome. This can be specifically seen in cases lacking timeline continuity. For example, a case report on hepatic hemangiomas described a spontaneous sudden regression of the lesion without justifying or supplying enough evidence as to how this change came to be or was assessed [34]. In addition to bias concerns, case reports and series are highly susceptible to confounding, as individualistic characteristics of patients could vary from history to presentation to treatment response and follow-up adherence; thus, generalizing findings from SRs of cases should be done with caution. External validity evolves from properly randomized and controlled evidence, which is not available in data pooled from case reports. In a SR of case reports, we propose the following guide for authors and reviewers assessing the generalizability of their findings:

1. Sample size: Since data in a SR of cases cannot be random, a larger sample size could partially compensate for population representation. A larger sample size can allow for more confident extrapolation of results. While a limited number of included cases may not be as representative of a sample. In this regard, subgrouping and stratification of cases can also prevent potential inaccurate generalization.
2. Method characteristics: Following the previous point, authors should carefully design their inclusion criteria, attempting to broaden the scope and add diversity, while confining their inclusion to the clinical condition under study. A broader scope can increase the power of the review but could distort vision from the target in question. Thus, a balanced inclusion criterion can strongly influence the generalizability of findings.
3. Threats and supporters: Certain factors can directly impact study’s generalizability, for instance, a biased or unbalanced selection of reports greatly affects the confidence in results representation of the actual population. Similarly, situational inclusion -like COVID-19 vaccination patients- cannot be easily generalized to similar yet situationally different populations -like patients receiving other vaccines.
4. Replication potential: A good measure of potentially generalizable findings is an assessment of its replication potential. If a certain methodology can be easily adopted in varying circumstances, and if it generates reproducible evidence, generalizing its findings can be attempted more confidently.

Assessing the quality of case reports or case series is a limited and difficult process, and while the quality of clinical trials (for example) has been extensively investigated and standardized, case reports’ quality is less addressed. Three main quality assessment criteria for case reports/series are available; the first is the CARE checklist mentioned previously [13], the second is JBI’s critical appraisal tool [8], and the National Institute of Health’s (NIH) quality assessment tool [35]. JBI and NIH tools are more suited for case report quality assessment post-publication. Both tools try to assess methodological rigor through questions on clear reporting and detailed description. Despite both tools being in a checklist format, they both do not provide a quantitative score for case quality, but rather provide a qualitative assessment. Both tools provide acceptable quality assessment criteria; however, the NIH tool features some broad and subjective questions that are directed towards a widened base of situations, in contrast to JBI’s assessment tool, which is more precisely tailored towards case reports and case series. JBI’s quality appraisal tool also directly addresses the rarity and relevance of the report, especially for case series, which could help in guiding the evidence to higher quality. It is worth noting that some authors tend to use individualized or modified quality appraisal tools, which could be justified, but the use of standard quality appraisal tools is recommended. A comprehensive comparison between JBI’s, NIH’s, and CARE checklist tools is provided in Table 1.

**Table 1:** Comprehensive comparison of NIH, JBI, and CARE checklist tools for quality assessment

Item/tool	NIH	JBI	CARE checklist
Definition	In 2013, NHLBI developed a set of tailored quality assessment tools to assist reviewers in focusing on concepts that are key to a study’s internal validity. The tools were specific to certain study designs and tested for potential flaws in study methods or implementation. Consists of 9 items for case series and 6 items for case reports	Developed by the JBI and collaborators and approved by the JBI Scientific Committee following extensive peer review. It aims to include a summary of the best available evidence. Consists of 8 items for case reports and 10 items for case series	Developed by an international group of experts to support an increase in the accuracy, transparency, and usefulness of case reports. Consists of 13 items and help writer to write a well-structured case report
Advantages	1. Scoring: Help to give a quality score for each case 2. Clear thresholds: dividing score into 3 grades (Good,” “Fair,” or “Poor”) based on the score from 9 3. Covers all quality aspects of the case: selection of participants into the study bias: questions 2, 3 and 4, measurement of outcomes bias: questions 6 and 7, selection of the reported results bias: 9, missing data bias: question 7 and 9, confounding & other methodological bias: 1, 8 and 5	4. Comprehensive: Include Separate checklists for case reports (8 items) and case series (10 items) 5. Scoring: Help to give a quality score for each case 6. Covers all quality aspects of the case: Bias in selection of participants into the study: questions 1, 4 and 5, bias in measurement of outcomes: questions 2 and 3, bias in selection of the reported results: questions 6 and 7, and bias due to missing data: question 8	Help to Standardized reporting of case reports and works as a reporting guideline designed to ensure all key elements of a case report are included
Disadvantages	1. No specific tool for case reports 2. Numeric outcomes maybe not cover or skip the subjective outcomes that can not be measured	1. Time consuming 2. No numeric score	1. Time consuming 2. Can not be used as tool to score 3. Do not cover case series

## Steps to conduct a SR of case reports

1. Finding the idea (Fig. 1, step 1)

**Fig. 1:**
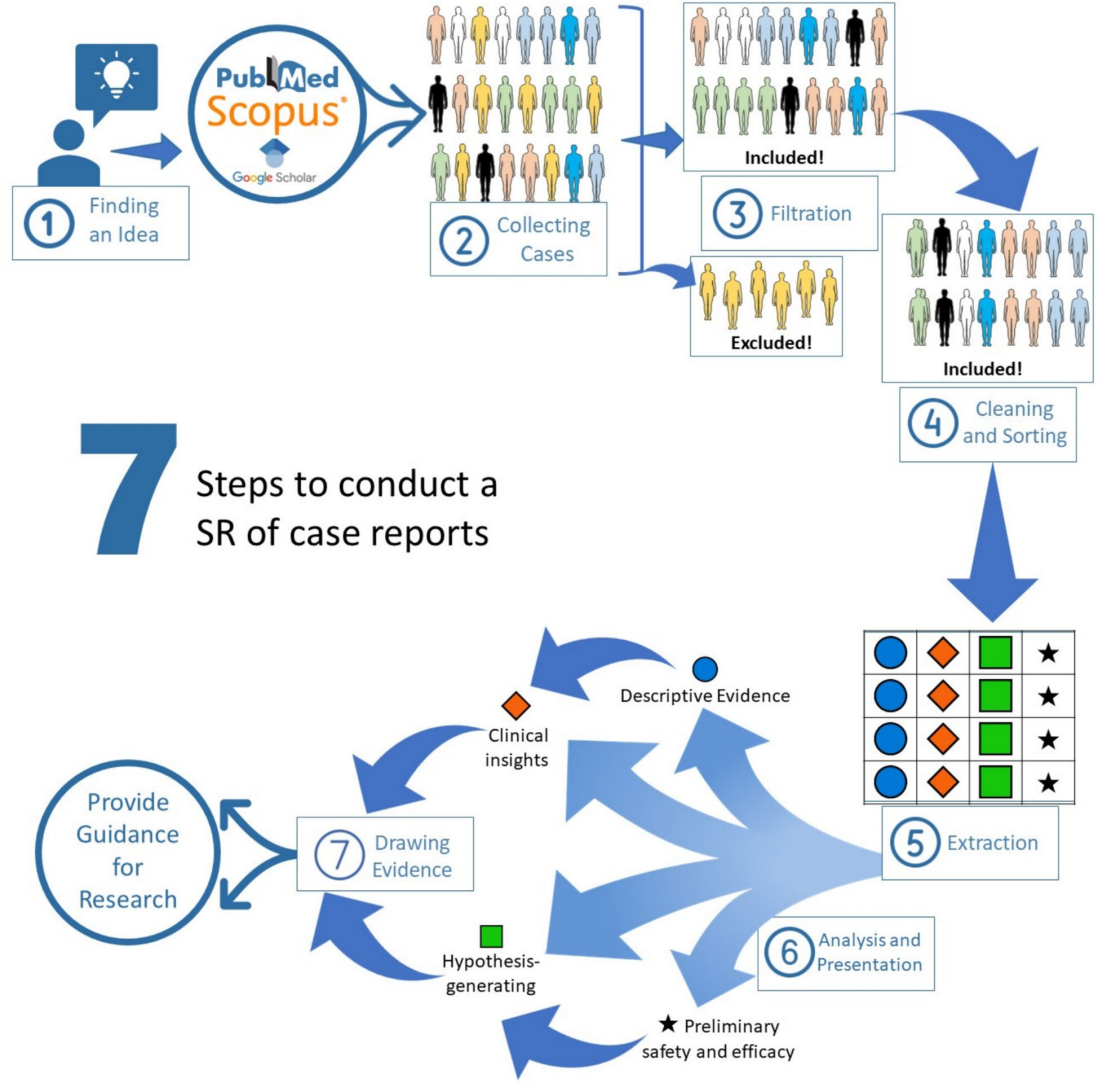
Steps of conduction a SR of case reports
  The first step in this type of research is to find a valid idea. There are different methods to find ideas that could be suitable for the SR of the case reports. The first method is to focus on a rare disease or complication that is reported in the literature as a case report or case series with minimal or no RCTs or observational studies due to the rarity of this disease or this complication. Other methods include gaps in knowledge, where we need to cover specific areas about some diseases or side effects that are only reported in case reports and case series.
2. Literature review (Fig. 1, step 1)
  Before writing about any topic, the author should establish the need for this paper. There is no need for more duplicated papers. A literature review helps the author understand the uncovered area of the idea. It is a document that describes your project’s primary and secondary sources, plus how they relate to your hypothesis. Also, it demonstrates how existing knowledge can serve as a foundation for your research, explains how your project may provide a more detailed evaluation of an issue, and structures your sources using a specific strategy [36].
3. Develop a PEO/PIO question (Fig. 1, step 1)
  PICO is usually used in evidence-based practice to frame and answer a clinical question. PEO and PIO are the most common research forms used in the SR of the case reports. Where P refers to population, I for intervention, C for comparator, E for exposure, and O for outcome [37, 38]. After an idea has been identified, it is wise to prepare a research protocol and to register it by a trusted platform like PROSPERO. Although these platforms typically target higher-level reviews, they can improve the transparency and credibility of the review [39].
4. Search the databases (Fig. 1, step 2)
  According to the AMSTAR guidelines, two databases should be searched in the SR/MA at the minimum, but more databases result in more coverage for all papers. Here, we propose six databases (PubMed, Scopus, Web of Science, EMBASE, Cochrane, and Google Scholar) [40]. Developing keywords is done according to the PEO/PIO question. The keywords of each item are added to each other by OR, while the keywords of different items are added by AND [41]. Using MeSH terms and Boolean operators in developing the search strategy can significantly impact the covered scope. For example, using the following search query: *[“COVID-19 vaccination”, “GBS”]*, can easily miss significant articles that may be relevant to the search. On the contrary, using this search query: *[((((((COVID-19 vaccination) OR (COVID-19 vaccination[MeSH Terms])) OR (coronavirus vaccine)) OR (coronavirus vaccine[MeSH Terms])) OR (mRNA vaccine)) OR ((mRNA vaccine)[MeSH Terms])) AND ((((Guillain-Barré syndrome) OR (Guillain-Barré syndrome[MeSH Terms])) OR (GBS)) OR (GBS[MeSH Terms]))]* would include all relevant articles without overlooking crucial research [42].
5. Studies filtration according to eligibility criteria (Fig. 1, steps 3 and 4)
  Filtration process is done at two levels: first level at the title and abstract level and the second level at the level of full text. The selection of the retrieved articles for further assessment is based on eligibility criteria which are based on the PIO/PEO questions and study design to reduce the chance of including unrelated studies. According to a study conducted by Tawfik et al. [43], at least three reviewers should work independently to reduce the chance of error, particularly in teams with a large number of authors to add more scrutiny and ensure proper conduct. Before any screening, duplication removal will be done by Endnote, and after this step, duplication removal will be done manually if it is detected. Then the second level is done after full text downloading and screening, and similarly, three reviewers work independently to decide on included full texts according to eligibility criteria and according to the PRISMA checklist [44]. Exclusion criteria should be written at the full text level [45]. Eligibility criteria should be clear and focus on the diagnostic criteria of the cases. The steps conducted during this stage can be easily and clearly summarized using a PRISMA chart, where the stages of search, first and second filtration, and exclusion criteria can be clearly elaborated. Excluding case reports can rely on many factors, especially lack of clinical information or unclear diagnostic methods [44]. An example of the filtration process figure is provided in Fig. 2, adopted from Abuawwad et al. [28]. Integrating the PRISMA flow chart with Fig. 1 can help the author to understand the concept and steps of the SR of case reports more. Step 2 in Fig. 1 matches the identification step of the PRISMA chart; step 3 equals the screening step in the PRISMA; and step 4 in Fig. 1 corresponds to the included phase in the PRISMA chart.

**Fig. 2:**
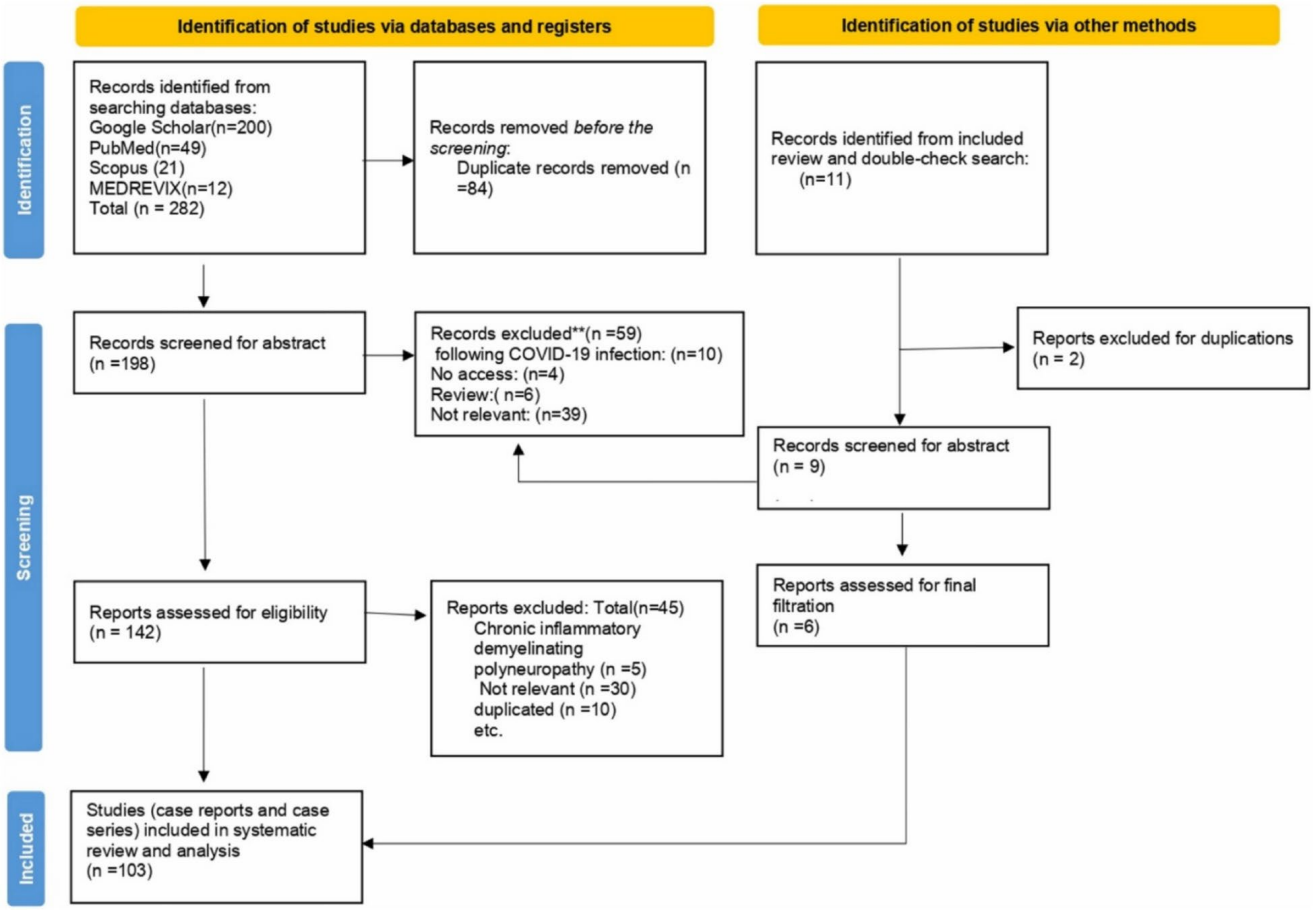
PRISMA chart summarizing the filtration process for Abuawwad et al
6. Data extraction and quality assessment: (Fig. 1, step 5)
  The PICO question, objectives, and hypothesis of the research should all be taken into consideration when extracting relevant information from cases. Data extraction and reporting should be performed in compliance with the 2013 CARE checklist [13]. There are numerous approaches to extracting the data. Excel and Review Manager are two of the most often utilized programs. While extraction sheets vary from one study to the next, several lines are constantly appropriate to research. These lines are the characteristics of the paper, including the study type, first author’ last name, sample size, publication year, and study title; the characteristics of the included patient, including age, gender, occupation, residency, special habits of medical importance, and marital status; the associated common comorbidities, such as diabetes and hypertension; and the quality assessment of the paper according to the tool used. However, after these parts are modified according to the research aims, the other sections, which differ from one paper to another, should be developed. As for quality assessment, NIH or JBI tools can be applied with ease, and each included case should be assessed individually and objectively.
  One challenge that researchers may face is the lack of information or missing-data in reviewed case reports. A few measures could be used to overcome this issue, the first of which is to leave the data as is, without intervening. This should be reflected in any conducted analysis or data reporting, since it might affect the extent to which reviewing was successful. Another technique is to use analytical missing data imputation, in an effort to prevent data shrinkage. This technique must be used with care, since it is greatly dependent on missingness mechanisms and model sensitivity. Using missing data imputation supports the integrity and rigor of included cases, but its use requires experience and comfortability with statistical analysis software. A third method that can be used to make up for missing data is to contact the journal or authors of cases with missing data, in an attempt to collect required information for data fulfillment.
  According to the research’s PIO/PEO, a comprehensive extraction portion for each item is prepared as follows. (We only provide a few instances; however, these examples should be modified in accordance with the research PIO/PEO.) Table 2 illustrates an example of data extraction in SRs of cases.

**Table 2:** Example for data extraction form

Item	Item from example	Items to extract
Population	Patient who received COVID-19 vaccination.	The main concerns were age, sex, occupation, marital status, systemic disease, COVID-19 testing, COVID-19 vaccine type, and dose.
Intervention/exposure	Development of GBS.	The main concerns were motor, sensory, and autonomic affections; the duration between vaccination and the development of symptoms; the distribution and severity of weakness with MRC score details; investigations for GBS (CSF); routine blood tests; and nerve conducting studies Also, we extract data about the diagnostic method based on Brighton criteria.
Outcome	Prognosis of the patients.	The main concerns were the main management and the patient’s result if the patient becomes an independent ambulant, respiratory compromise, bulbar palsy, and mortality.
7. Data analysis (Fig. 1, steps 6 and 7)
  Data extraction will be followed by data cleaning and coding in order to begin the analysis. Numerous software programs, such as SPSS [46], R program [47], Jamovi [48], Comprehensive Meta-Analysis Software [49], and many more, can be used for data analysis. Typically, descriptive and inferential statistics are used to analyze the data in the SR of the case reports. The descriptive statistics are classified as follows: mean with standard deviation for numerical data that is regularly distributed and median with interquartile ranges for data that is abnormally distributed. Additionally, frequency and percentages will be utilized for categorical data. In cases where rare data is collected, basic comparative statistics may be utilized. Comparing subgroups of included cases can provide crucial insight if data allows. For example, a logistic regression linking clinical factors to prognosis can improve insight into case progression or remission. Similarly, meta-analysis of proportion or meta-regression can be utilized to look into demographic variables that influence certain symptoms or adverse events. Parametric and non-parametric tests are the most often utilized tests at the inferential statistical level. Additional analysis, including Cox regression and survival analysis, can be performed. Figure 1 provides an illustration of the steps of conducting a SR of cases.

## Case study

In an attempt at understanding the importance of SR of cases, we will analyze an article that followed this method and point out what it achieved in terms of evidence generation.

A great example of a SR of case reports is the review conducted by Abdel-Wahab et al. on case reports of antiphospholipid syndrome following infection [19].

The authors of this article made a great effort to formulate their manuscript. Their main conclusion was an indication that an elevation of aPL antibodies, manifesting antiphospholipid syndrome (APS), has been observed with various types of infections.

Until the date of this writing, the Abdel-Wahab et al. article had been cited 513 times according to Google scholar. Several articles of various methodologies addressed the same issue later, and in 2023, a review article on the pathophysiology of APS considered viral and bacterial infections as potential etiologies for APS, citing several articles, including a meta-analysis [50]. This SR of case reports highlights the practicality of this methodology, as it investigates a rare occurrence, for which higher level evidence is difficult to acquire, and a potential insight could be drawn for future reference. In simple terms, the SR of cases performed by Abdel-Wahab et al. helped carve the path for future preference, and with the emergence of COVID-19, cases of APS were observed in association with the infection, and articles investigating this phenomenon used Abdel-Wahab’s paper for insight on several aspects [51, 52]. Table 3 summarizes the case study described above.

**Table 3:**
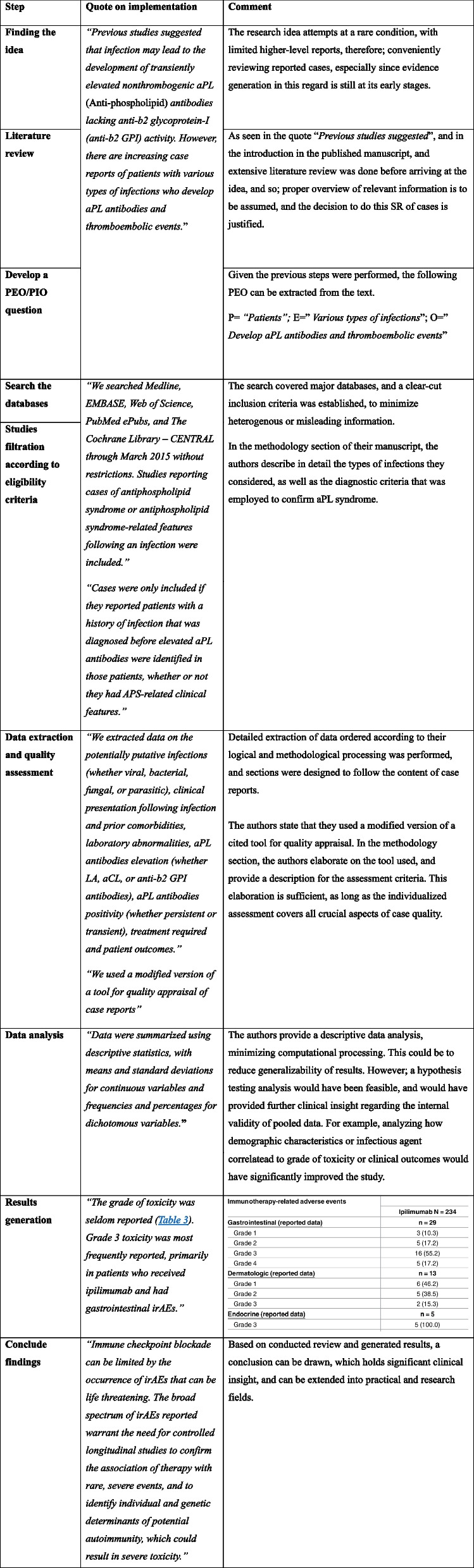
A case study of implementation of a SR of cases

## Discussion

Case reports and case series are two of the main observational studies that report rare diseases or unexpected events. The SR of case reports is a tool to collect all these cases in one pool that helps us draw evidence about the patients’ characteristics, complications, risk factors, and prognosis. This paper aims to provide a description and step-by-step guide to the SR of case reports and case series.

The SR and meta-analysis of randomized control trials provide the highest possible evidence in the hierarchy of evidence [6]. However, for rare diseases, side effects, or unexpected events, conducting an RCT is impossible and cannot be done due to the rarity of patients. On the other hand, the SR of case reports and case series covers this gap in knowledge and provides the researcher with clear and significant lines that can help them conduct interventional studies based on the paved road of the cases. In addition, we suggest to readers how they can develop this type of research with many examples and a full case study that helps them understand these steps. We hope that researchers could gain benefit from this study, and they can start to conduct SR of the case reports based on this work.

Some challenges may arise when we conduct a SR of case reports, such as incomplete data or missing some data from the cases. In our published SRs of cases, these challenges were dealt with as described above. For example, missing data was handled analytically or by author contact, and in difficult cases, they were blankly reported and pointed at as such. Similarly, contradicting reports were sometimes encountered; therefore, findings were developed depending on pooled results rather than individual reports.

Finally, we suggest that there is a need to develop a specific protocol to assess the quality of the SR of case reports and case series in order to help policy makers, managers, and other decision makers formulate appropriate recommendations for practice or policy and improve the quality of this type of methodology [44]. Also, there is a clear need to conduct a direct comparison between the results of the SR of case reports and the results of other observational and interventional studies after conducting it in order to highlight the differences, limitations, weaknesses, and strengths of the SR of case reports.

## Conclusion

Systematic reviews of case reports provide an important tool for the purpose of researching rare or unexpected events, and a clear methodology with a step-by-step guide is suggested in this article. The findings from the SR of case reports about rare diseases, drug side effects, or particular surgical procedures can be used by authors and clinicians to forecast the occurrence of these events, prevent them before they happen, or appropriately treat them in the event that prevention is unsuccessful. Additionally, the SR of case reports can provide a foundation for greater strength study on adverse effects or rare diseases. Concerns regarding the quality of case reports are in need of further assessment, and a global tool for this purpose is highly in demand.
